# Chemical evidence of rare porphyrins in purple shells of *Crassostrea gigas* oyster

**DOI:** 10.1038/s41598-020-69133-5

**Published:** 2020-07-22

**Authors:** Michel Bonnard, Sonia Cantel, Bruno Boury, Isabelle Parrot

**Affiliations:** 1 IBMM, Univ Montpellier, CNRS, ENSCM, Montpellier, France; 2 TARBOURIECH-MEDITHAU, Marseillan, France; 3 ICGM, Univ Montpellier, CNRS, ENSCM, Montpellier, France

**Keywords:** Chemical biology, Evolution, Environmental sciences, Ocean sciences, Chemistry, Materials science

## Abstract

The colour of oyster shells is a very diverse characteristic morphotype, forming intriguing vivid patterns both on the inside and outside of the shell. In the present study, we have identified for the first time, the presence of several porphyrins as constituents of the shell pigmentation of the *Crassostrea gigas* oyster consumed worldwide. The precise molecular structures of halochromic, fluorescent and acid-soluble porphyrins, such as uroporphyrin and turacin, are unambiguously determined by reverse phase liquid chromatography combined with high resolution mass spectrometry. Their presence account for the purple colouration of shells but also for the dark colouration of adductor muscle scars. We have also defined the endogenous origin of these porphyrins, specifically secreted or accumulated by the shell forming tissue. These findings are pioneering analytical proofs of the existence of the haem pathway in the edible oyster *Crassostrea gigas*, evidenced by the chemical identification of haem side-products and supported by the recent publication of the corresponding oyster genome.

## Introduction

Shells are the armour of many molluscs. Their diversities of shape, ornamentation and colour have always attracted humans throughout the ages. In bivalves, related to the requirements of the pearl farming industry, shell morphotypes and colours of pearl oysters (e.g. *Pinctada margaritifera*) have been extensively studied compared to their edible cousins (e.g. *Crassostrea gigas*)^1^. Prized by gourmets, the aesthetic appeal of shellfish as a gastronomic showcase is arousing growing interest. However, the accumulation of wasted shells becomes a real environmental problem, predominantly in oyster farming or tourist areas^1^. At a time when sustainable and intelligent uses of renewable aquatic resources are needed, the transformation of this calcareous by-product into a value-added material is a relevant challenge to be taken up.

While the use or transformation of calcium carbonate or its organic matrix is the first-line source for recycling edible oyster shell, its colouration is still unwell understood and therefore little valued^1^. Despite the functional roles of shell colours in camouflage, thermoregulation or immunity, the molecular mechanisms related to shell colouration remain poorly documented^2^. This gap of knowledge is partly due to the lack of elucidation of the chemical nature of shell pigments, particularly in the *Crassostrea* genus. By combining the identification of shell pigments with corresponding biosynthetic pathways and associated genes, key information on the relation between shell colouration, mineralization or other molecular mechanisms can be obtained as previously reported in marine snails^3^. Therefore, the identification of shell pigments appears to be a possible gateway to the recovery of natural dyes from wasted oyster shells for a broad range of applications in health or material science, helping to contribute to a sustainable future for shellfish farming industry^1^.

In the Pteriomorphia group, including edible and pearl oysters, the distribution of shell colours has been classified by Grant et al*.*^4^ in eight phenotypes, varying from yellow to orange, red, blue, green, purple, brown and black, more or less associated together, the brown and black colours being often assigned to melanins with poor analytical evidences. Besides, shell colours can result either from the interference of diffracted light over nanostructured material (structural colours), or from pigments such as melanins, tetrapyrroles and carotenoids, or from a combination of both^2^. This is particularly the case of highly prized shells and pearls of *Pinctada margaritifera* where the iridescent nanostructure of mother-of-pearl is often associated with a fluorescent cyclic tetrapyrrole^5,6^. Since the early work of the pioneer A. Comfort postulating on the basis of paper chromatography and ultraviolet spectroscopy that uroporphyrin is the major pigment deposited in pearl oysters shells^7,8^, little is known about the chemical structure of oyster shells pigments.

Compared to the shell of pearl oysters (e.g. *Pinctada maxima*), the shell of the edible oyster *Crassostrea gigas* has a distinct calcareous microstructural layout with an adductor muscle scar (AMS) made of aragonite and frequently coloured^1,4^. Only this small coloured part of the inside shell has been studied for pigment identification^9^, the other coloured shell patterns totally lacking of in-depth chemical studies. In this particular case, eumelanin has been proposed to contribute to the AMS black colouration, but its chemical identification is questionable. Indeed, as a consequence of its low solubility, the chemical investigation of its structure was only based on limited spectroscopic data (infrared and UV spectroscopy). In this regard, the recent investigation of *C. gigas* dark AMS by mass spectrometry has demonstrated the absence of melanins markers usually obtained after alkaline oxidation (pyrrole-2,3-dicarboxylic acid, pyrrole-2,3,5-tricarboxylic acid, thiazole-4,5-dicarboxylic acid and thiazole-2,4,5-tricarboxylic acid)^10^. Identifying pigments associated with black and other frequent shell colours (e.g. yellow, red and purple) would constitute a major breakthrough, shedding light on their origin and function. Recently, Feng et al*.*^11^ have evidenced the key role of different expressed long non-coding RNA and mRNA transcripts potentially associated with mineralization and shell pigmentation, among which 6 mRNAs are identified to influence the biosynthesis of pigments including melanins, carotenoids, tetrapyrroles, and ommochromes. However, the occurrence of these pigments in *C. gigas* oyster shells remains to be established, as for many other molluscan shells.

In this study, we present evidence of the nature and origin of *C. gigas* shell purple colours through photophysical and chemical investigations. This Japanese oyster, introduced in France on large scale, usually displays pink, bright- and dark-purple patterns on its shell, with a natural aestheticism and a marked AMS. In order to distinguish colours due to a structure or pigment, shell purple patterns and dark AMS were first investigated by scanning electron microscopy and compared to white patterns as negative controls. A group of tetrapyrroles was then identified on the basis of photophysical properties of samples dissolved in acid. Their unambiguous identification in shell purple patterns and dark AMS was demonstrated by reverse-phase liquid chromatography combined with high-resolution tandem mass spectrometry (RPLC-Q-ToF-HRMS/MS). The constant progress made in HRMS combined with adequate separation by RPLC appears as the gold standard method for the unambiguous identification of biomolecules from complex natural samples and more specifically porphyrins from molluscan shells, as recently demonstrated by Verdes et al*.*^12,13^. Additional chemical investigations conducted on dark secretions of the mantle allowed to highlight the presence of uroporphyrin. All together, these findings confirm the endogenous origin of tetrapyrroles found in shell purple patterns. These discoveries support the identification of specific genes of the haem pathway, leading to a better overall understanding of the pigmentation process in *C. gigas*. It also gives interesting opportunities for shell harnessing as a by-product.

## Results

### Morphology and structure of shell purple patterns and dark adductor muscle scar (AMS) of *C. gigas*

**Figure 1:**
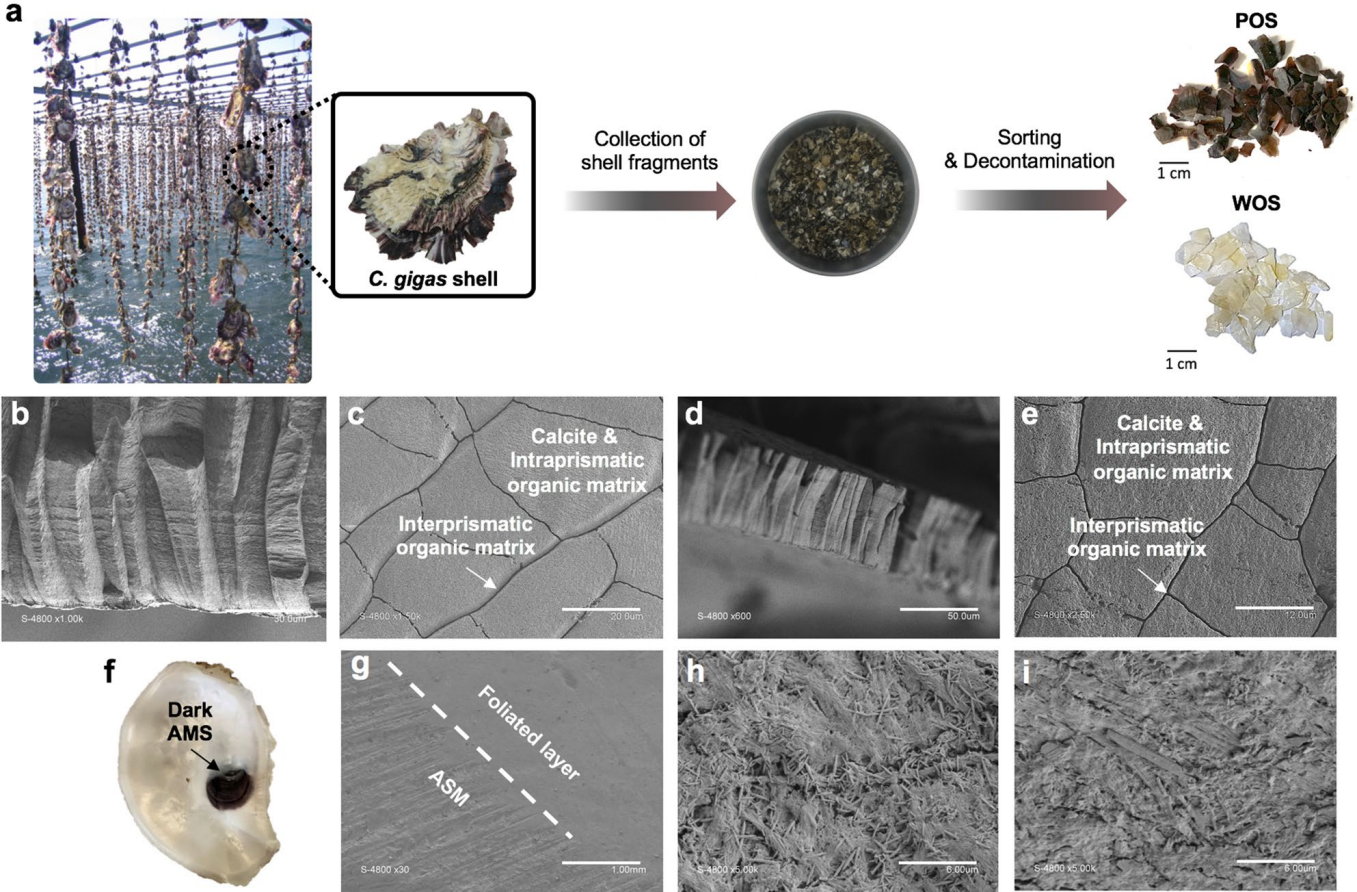
Schematization of shell samples collection, preparation and structural characterization by SEM. (**a**) Schematic figure of decontaminated WOS and POS collected from adult living *C. gigas* oysters (Thau lagoon, France). (**b,c**) Characteristic SEM images of decontaminated WOS (cross-section and top view, scale bar = 30 and 20 μm, respectively). (**d,e**) Characteristic SEM images of decontaminated POS (cross-section and top view, scale bar = 50 and 12 μm, respectively). (**f**) Representative photograph of a decontaminated dorsal valve with a dark adductor muscle scar (AMS), obtained from adult *C. gigas* wasted shells. (**g–i**) Characteristic SEM images of decontaminated dark AMS (top views, scale bar = 1 and 6 μm).

With the ambition to provide complete information on the nature of colours of *C. gigas* shell and AMS (pigment or structural colour), we first conducted a visual description of oyster shells at different stages of development from juvenile to adult, collected from different location (Supplementary Fig. S1). In general, epibionts and brown organic periostracum cover the outer shell colours (Supplementary Fig. S1k)^4,14^. The coloured patterns found in *C. gigas* shells covered shades from pink to dark-purple and yellow–brown over a whitish background layer (Supplementary Fig. S2a-b). Purple patterns were random distributions of irregular developing bands and radiating sectors of variable widths (Fig. 1a). No obvious morphological or macro-structural differences could be observed compared to white patterns. In most cases, AMS were dark (Fig. 1f and Supplementary Fig. S1l), potentially black or purple-black, with no systematic correlation with the colour of the outer calcified layer.

Although not identifiable by naked eyes, we speculated on a possible structural colour related to purple patterns and dark AMS at micro- or sub-micrometre scale. While the layout of *Pinctada* spp. shells is made of an inner iridescent nacreous and an outer prismatic layer often associated with melanins deposition^15,16^, the calcitic shell of *C. gigas* consists of a prismatic outer layer and a foliated inner layer more or less interrupted by a chalky structure (Supplementary Fig. S2)^1^. Consequently, we also hypothesized that a deposition of pigments could be associated with the formation of a specific microstructure. For this purpose, epibionts (e.g. diatoms, rhodophytes or chlorophytes) and periostracum that may contribute to orange, red, blue, green and brown colours^4,14^, were removed by decontamination. Scanning electron microscopy (SEM) was then applied on white oyster shell fragments (WOS), purple oyster shell fragments (POS) and dark AMS. Both WOS and POS were constituted by the typical columnar prismatic structure of edible oyster shells (Fig. 1b–e), known to be the richest in organic matrix among microstructures occurring in molluscan shells^17^. At microscale, no specific structure-colour relationship could be highlighted. In contrast, dark AMS had specific networks of fibres, needles and pores (Fig. 1f–i). This structural arrangement was clearly different from the columnar prismatic structure of WOS and POS. At this stage, we could hypothesize that POS colouration might be due to the deposition of one or more pigments, as no microstructural differences have been detected with the negative colour control (WOS). In contrast, it is more difficult to be as affirmative concerning dark AMS with a different microstructure.

### Solid-state fluorescence of shell purple patterns and dark AMS of *C. gigas*

In contrast to iridescence which is related to the nanostructure of nacreous layers, fluorescence in molluscan shells is generally attributed to fluorescent tetrapyrroles like bile pigments and porphyrins^2^. The latter are highly fluorescent in solution, but in solid state, their aggregation leads to fluorescence quenching, a well-documented property resulting from coplanar π-conjugated stacking^18,19^. Complexation with proteins may also result in fluorescence quenching^20^. In the Pteriomorphia group, limited to oysters, the emission of bright pink-red fluorescence has only been reported from the shell of *Pinctada vulgaris* pearl oyster exposed to UV light at approximately 400 nm^21^. In our study, under various monochromatic lights (λ ~ 254, 365 and 400 nm), no fluorescence has been observed from WOS, POS and dark AMS in contrast to the dark-brown patterns on shells of *Pinctada radiata*.

### Identification of fluorescent tetrapyrroles deposited in shell purple patterns and dark AMS of *C. gigas*

**Figure 2:**
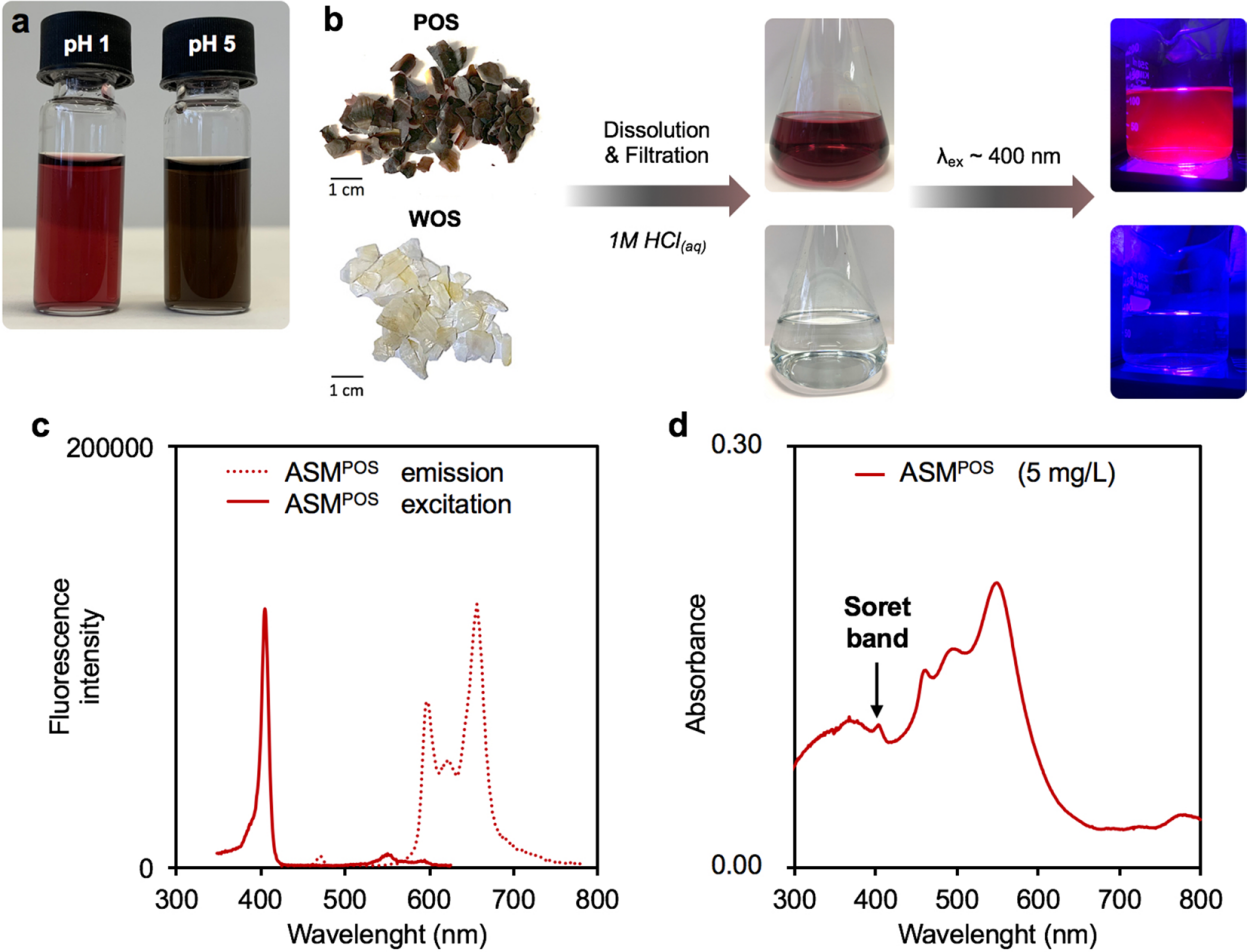
Photophysical properties of *C. gigas* shell samples. (**a**) Photograph of the halochromic or ionochromic property of decontaminated POS dissolved in 1 M aqueous HCl (pH ~ 1) and 1 M aqueous CH_3_COOH (pH ~ 5). (**b**) Schematization of the protocol for photoluminescence investigation of decontaminated WOS or POS, dissolved in 1 M HCl_(aq)_. (**c**) Excitation and emission spectra of ASM^POS^. (**d**) UV–vis absorption spectrum of ASM^POS^.

Considering the well-documented photophysical properties of bile pigments and porphyrins in solution^22^, we undertook to study the fluorescence and UV–visible absorption properties of acidic solutions of dissolved shell samples. The dissolution of decontaminated POS results in a purple-red solution in 1 M aqueous hydrochloric acid and purple-dark in 1 M aqueous acetic acid, revealing an interesting halochromic or ionochromic property of acid-soluble pigments (Fig. 2a). In this hydrochloric acid solution, the colourless interprismatic organic matrix was isolated and examined by SEM to observe its polygonal structure, as previously reported in the literature (Supplementary Fig. S3).

The general photophysical behaviour of dissolved POS was also observed on solution obtained by dissolution of dark AMS. In contrast, the dissolution of WOS resulted in a colourless solution (Fig. 2b). At this point, we could sustain that colours of shell purple patterns and dark AMS are not the result of a specific meso-structuration but are due to the deposition of acid-soluble pigments. The halochromic or ionochromic property of such shell pigments is fully compatible with bile pigments and porphyrins^23^.

After dissolution and filtration, solutions of decontaminated WOS, POS and dark AMS (commonly referred to acid-soluble matrices in literature ASM^WOS^, ASM^POS^ and ASM^AMS^, respectively), were exposed to a monochromatic light at λ ~ 400 nm (Fig. 2b). Unsurprisingly, no photoluminescence was emitted by ASM^WOS^. On the opposite, a bright pink photoluminescence was observed from ASM^POS^ and ASM^AMS^, a behaviour also fully compatible with bile pigments and porphyrins. The emission spectrum of ASM^POS^  at λ_ex_ = 405 nm has a three-peak profile in the 550–750 nm range (Fig. 2c), similar with those of carboxylic acid porphyrins already reported in the literature from natural and biological samples^24,25^. Such photoluminescent emission, only observed from acid-soluble matrices of coloured samples (ASM^POS^ and ASM^AMS^), supports the presence of fluorescent tetrapyrroles in shell purple patterns and dark AMS.

UV–vis adsorption spectroscopy provided additional information (Fig. 2d). The ASM^WOS^ did not absorb in the visible region (background signal). The shoulder observed at 404 nm in the ASM^POS^  absorption spectrum matched the Soret band of fluorescent carboxylic acid porphyrins described in the literature, such as uroporphyrin. The supplemental absorption bands at 464, 496 and 552 nm with higher intensities than the Soret band at 404 nm could be explained by multiple factors. First, a mixture of different types of porphyrins (free-base or metaled) may have different Soret band and Q-bands values. Second, aggregations of porphyrins due to the ionic strength of ASM^POS^ containing Ca^2+^, are well known to form additional absorption bands in visible region and/or to increase/decrease the Soret band intensity^22,26^. Third, the presence of multiple absorbing species associated with porphyrins by complexation and/or ionic interactions, may lead to superposition of individual absorption spectrum as already observed with mixtures of melanin and porphyrin^27^. However, the absorption spectrum of ASM^AMS^  is less exploitable, certainly due to the low concentration of absorbing species (Supplementary Fig. S4b). Only the absorption bands at 496 and 552 nm are observed in the visible region. Despite the emission of photoluminescence under λ_ex_ ~ 400 nm, the Soret band is not observable.

### Identification of uroporphyrin deposited in shell purple patterns of *C. gigas*

**Figure 3:**
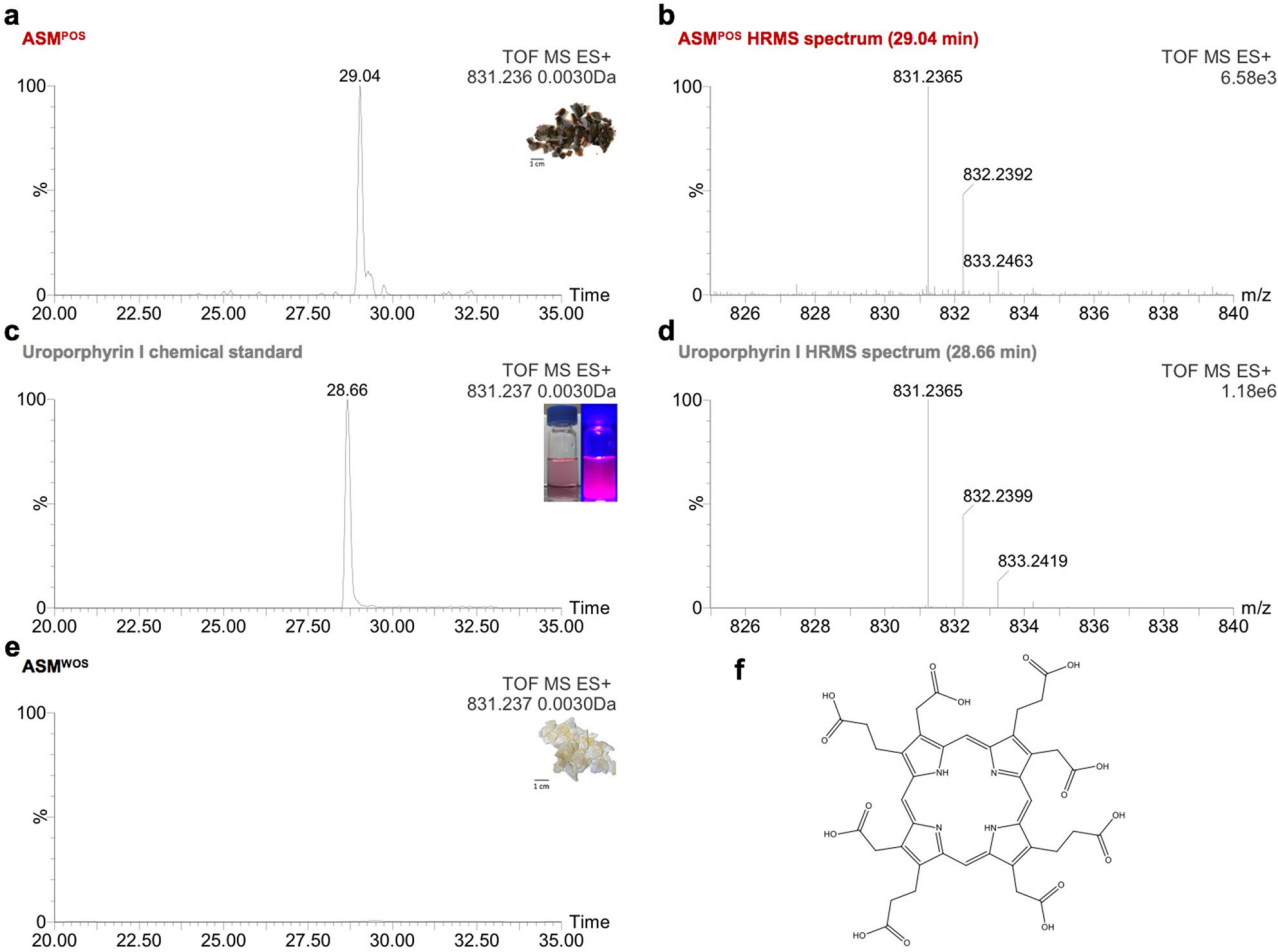
Chemical investigation by RPLC-HRMS of ASM^POS^ and ASM^WOS^ in comparison with a commercial standard of uroporphyrin I. (**a–d**) Chromatograms of extracted uroporphyrin I or III ion detected in ASM^POS^ and chemical standard of uroporphyrin I with the corresponding high resolution mass spectra. (**e**) Chromatogram of extracted uroporphyrin I or III ion undetected in ASM^WOS^. (**f**) Chemical structure of uroporphyrin I (*m/z* calculated [M + H]^+^  = 831.2361).

Reverse phase liquid chromatography (RPLC) combined with high resolution mass spectrometry (HRMS), a method recently applied by Verdes et al*.* to identify molluscan shell pigments^12^, is a powerful tool to access molecular formula of ionizable pigments, even at trace amount level. In order to precisely identify fluorescent acid-soluble tetrapyrroles specifically deposited in shell purple patterns of *C. gigas*, the chemical investigations of ASM^POS^ and ASM^WOS^ as a negative control, were conducted by RPLC-HRMS in electrospray positive ionization mode. It has been described that in natural shell samples, only the isomeric type I and type III of carboxylic acid porphyrins can be found^20^. The molecular ion of uroporphyrin I or III was detected in ASM^POS^ (*m/z*_exp_ 831.2365 ± 0.0030, retention time of 29.04 min, Fig. 3a,b). These data are in good agreement with the analysis of an uroporphyrin I chemical standard ([M + H]^+^ at *m/z*_exp_ 831.2365 ± 0.0030, retention time of 28.66 min, Fig. 3c,d). In contrast no signal corresponding to the expected *m/z* of uroporphyrin molecular ion could be detected in ASM^WOS^ (Fig. 3e,f), supporting the specific deposition of uroporphyrin I or III in shell purple patterns.

### Identification of uroporphyrin and heptacarboxylic acid porphyrin deposited in dark AMS of *C. gigas*

Based on our premise, we investigated the presence of fluorescent acid-soluble tetrapyrroles deposited in AMS. The previous UV–vis absorption analysis of ASM^AMS^ suggested that the concentration level of acid-soluble porphyrins might be below the detection limit in current conditions. In order to allow their detection and characterization by RPLC-HRMS, an innovative decalcification treatment was developed on ASM^AMS^ by calcium fluoride precipitation with hydrofluoric acid (HF). This treatment enabled to prepare a sample more concentrated in organic species (from initially 0.02 to 10 mg/mL). Its analysis by RPLC-HRMS in electrospray positive ionization mode has shown the detection of the expected uroporphyrin I or III molecular ion but also the detection of heptacarboxylic acid porphyrin I or III molecular ion (respectively *m/z*_exp_ 831.2362 ± 0.0030, retention time of 28.06 min and *m/z*_exp_ 787.2471 ± 0.0030, retention time 28.93 min, Supplementary Fig. S4c–f). To the best of our knowledge, these data provide a pioneering evidence for the deposition of uroporphyrin and derivatives in dark AMS of *C. gigas*.

### Identification and chemical confirmation of uroporphyrin and derivatives deposited in shell purple patterns of *C. gigas*

In order to identify other potential acid-soluble porphyrins deposited in shell purple pattern as hypothesized by the UV–vis absorption analysis of ASM^POS^, sample concentration was performed prior to RPLC-HRMS. This treatment was also achieved by calcium fluoride precipitation with HF.

This approach allowed to identify an entire set of carboxylic acid porphyrins which can be classified as major and minor porphyrins. On the basis of elution profile, calculated [M + H]^+^ *m/z* and exact mass of carboxylic acid porphyrins reported from the literature (including mixtures with isomeric and hydroxylated forms)^20,28–31^, the molecular formula of several acid-soluble porphyrins and their potential structures could be proposed (Table 1). By order of elution, major porphyrins were identified as uroporphyrin, heptacarboxylic acid porphyrin and turacin, with type I isomers always eluted before the corresponding type III (Supplementary Fig. S5a–j). To our knowledge, turacin, a copper-metallized uroporphyrin, was detected for the first time in a natural sample other than turaco feathers^32^. The isotopic distribution of turacin molecular ion (Supplementary Fig. S5i,j) supported the presence of Cu(II) by comparison with the relative abundances of ^63^Cu and ^65^Cu isotopes. More surprisingly, the molecular ion of Cu(II)-metallized heptacarboxylic acid porphyrin was also detected in the set of minor porphyrins (Supplementary Fig. S6e). This is the first case of detection of this porphyrin in a natural sample. Minor porphyrins were identified as hexacarboxylic and pentacarboxylic acid porphyrins, coproporphyrin and traces of hydroxylated and isomeric forms (Supplementary Fig. S6a–h).

**Table 1:** Major and minor carboxylic acid porphyrins detected in concentrated ASM^POS^ by RPLC-HRMS in electrospray positive ionization mode.

Major carboxylic acid porphyrins	Retention time (min)	Calculated exact mass (± 0.003 Da)	*m/z* observed [M + H]^+^	*m/z* calculated [M + H]^+^
**Uroporphyrin I & III**				
C_40_H_38_N_4_O_16_	28.40 & 28.69	830.2283	831.2377 & 831.2356	831.2361
**Heptacarboxylic acid porphyrin I & III**				
C_39_H_38_N_4_O_14_	29.33 & 29.58	786.2384	787.2458 & 787.2485	787.2463
**Turacin I & III**				
C_40_H_36_N_4_O_16_Cu	29.58	891.1422	892.1512 & 892.1498	892.1501

Minor carboxylic acid porphyrins	Retention time (min)	Calculated exact mass (± 0.003 Da)	*m/z* observed [M + H]^+^	*m/z* calculated [M + H]^+^
**Hydroxy-heptacarboxylic acid porphyrin**				
C_39_H_38_N_4_O_15_	28.23	802.2333	803.2430	803.2412
**Hydroxy-hexacarboxylic acid porphyrin**				
C_38_H_38_N_4_O_13_	29.00	758.2435	759.2573	759.2514
**Ketoacid heptacarboxylic acid porphyrin**				
C_39_H_36_N_4_O_15_	30.03	800.2177	801.2264	801.2255
**Hexacarboxylic acid porphyrin**				
C_38_H_38_N_4_O_12_	30.18	742.2486	743.2563	743.2564
**Cu(II)heptacarboxylic acid porphyrin**				
C_39_H_36_N_4_O_14_Cu	31.21	847.1524	848.1592	848.1602
**Pentacarboxylic acid porphyrin**				
C_37_H_38_N_4_O_10_	32.40	698.2589	699.2664	699.2666
**Coproporphyrin**				
C_36_H_38_N_4_O_8_	32.50	654.2690	655.2742	655.2768

The chemical structure confirmation of the major porphyrins was conducted through their MS/MS profile using tandem mass spectrometry. MS/MS spectra of uroporphyrin (Supplementary Fig. S7a), heptacarboxylic acid porphyrin (Supplementary Fig. S7c) and turacin molecular ions (Supplementary Fig. S7b)^30,33^.

Finally, purification of major porphyrins has been attempted to elucidate more precisely their isomeric forms by nuclear magnetic resonance spectroscopy. However, NMR experiments only allowed us to observe the characteristic protons chemical shift of COOH and NH of uroporphyrin I (Supplementary Fig. S8a,b, respectively) by comparison with the uroporphyrin I chemical standard (approximately − 4 and + 10 ppm, Supplementary Fig. S8d,e). We estimated that the identification of other porphyrin isomeric forms by ^1^HMR experiments would require the extraction and enrichment of porphyrins from at least one 100 kg batch of POS.

### Identification of uroporphyrin in the shell forming tissue of *C. gigas*

**Figure 4:**
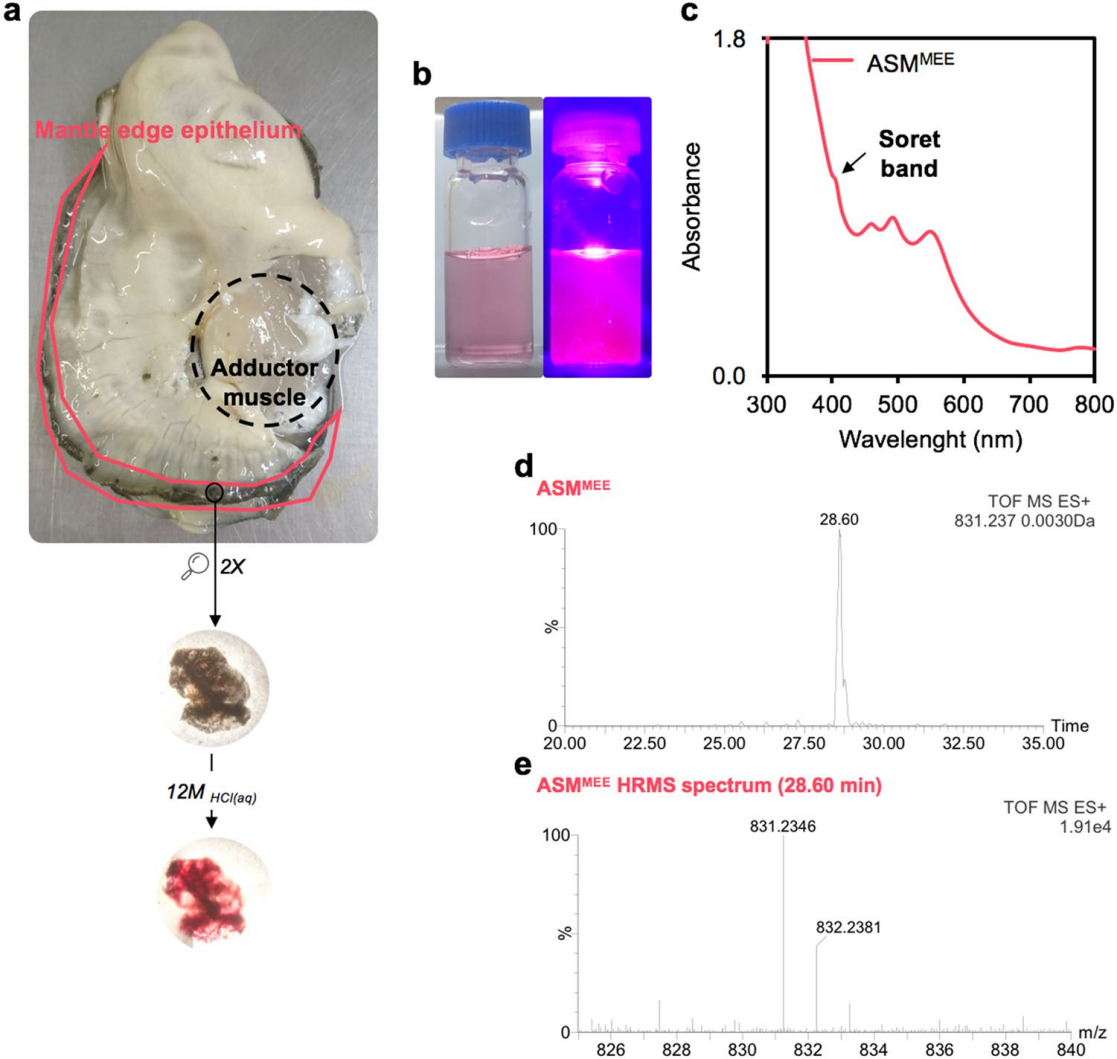
Identification of uroporphyrin in the mantle edge epithelium tissue (MEE) of *C. gigas.* (**a**) Schematic figure of brown-to-black secretions of the MEE, turning red in concentrated aqueous HCl. (**b**) Pink photoluminescence of ASM^MEE^ at λ_ex_ ~ 400 nm. (**c**) UV–vis absorption spectrum of ASM^MEE^. (**d,e**) Chromatogram of the extracted uroporphyrin I or III ion detected in concentrated ASM^MEE^ with the corresponding high resolution mass spectrum.

Upon observation, the light milky coloration of most of the organs was apparently unrelated to the coloration observed on the shell. However, viscera and mantle had a strong dark coloration which drew our attention to the possibility of a link with the colour observed on the shell. The mantle is usually coloured by brown-to-black secretions turning purple-red in contact with concentrated aqueous HCl (Fig. 4a), suggesting an important connection with shell pigmentations. In the general case of molluscs, the secretory cells of nerve fibres located in the mantle edge epithelium are believed to drive shell pigmentation, which is controlled by the neurosecretory system of the living animal^2,34^. In order to establish the origin of porphyrins-related shell pigmentation in *C. gigas*, the mantle edge epithelium (MEE) was collected from adult specimens (*n* = 10 animals), freeze-dried and subjected to immersion and agitation in 1 M aqueous HCl. The identification of porphyrins in the acidic extract (ASM^MEE^) was first suggested by its pink photoluminescence under a monochromatic light at λ ~ 400 nm (Fig. 4b). Furthermore, its absorption spectrum in the visible region is very close to the absorption spectrum of ASM^POS^ (a shoulder at 406 nm and supplemental bands at 464, 496 and 555 nm in Fig. 4c), suggesting a cross-relation between compositions of brown-to-black secretions of MEE and shell purple and dark patterns. In addition, the analysis of ASM^MEE^ by RPLC-HRMS has revealed the detection of uroporphyrin I or III molecular ion (*m/z* 831.2346 ± 0.0030, retention time of 28.60 min, Fig. 4d,e). These findings suggest that purple and dark patterns of the shell are composed of uroporphyrin and derivatives accumulated by the mantle of *C. gigas*.

### Identification of chlorophyll catabolites in viscera of *C. gigas*

Viscera, including intestine, are the only other purple or dark-coloured tissues of *C. gigas* (Supplementary Fig. S9a). In order to locate the origin of carboxylic acid porphyrins, the highly coloured soft tissues were collected from adult specimens (*n* = 10 animals). Viscera (VOT) were freeze-dried and subjected to immersion in 1 M aqueous HCl. The acidic extract (ASM^VOT^) emits a red photoluminescence under a monochromatic light at λ ~ 400 nm. Its absorption spectrum has a broad band at 417 nm and a weaker band at 667 nm (Supplementary Fig. S9b), which are characteristics of the Soret and Q-bands of chlorophyll a^35^. The ASM^VOT^ was further investigated by RPLC-HRMS and no signals corresponding to the carboxylic acid porphyrins ions previously identified in purple and dark *C. gigas* patterns or other known carboxylic acid forms were detected. On the other hand, molecular ions corresponding to chlorophyll catabolites were observed, forming characteristic dimer ions (Supplementary Fig. S9c–e)^36^. Chlorophyll catabolites certainly derives from the digestion of chlorophylls resulting from the algae-based oysters feeding. These findings converge towards the MEE-specific accumulation of carboxylic acid porphyrins responsible for purple and dark *C. gigas* shells colours.

## Discussion

The coloured patterns of molluscan shells were defined by C.-P. Nuttal and are still considered today to be *“distinct from irregular mottling …[and] consist[ing] of radiating sectors or rays of various widths, of concentric bands, of series of spots or blotches, of chevrons or zigzagging of complicated mosaics composed of geometrical (commonly triangular) figures of various sizes”*^4^*.* So far, shell colours was hypothesized to result from a periodic and repetitive deposition of pigment into the shell, a process achieved by the secretory cells of the mantle edge epithelium^37^. In our study, we observed that the purple patterns over *C. gigas* shells were characterized by a random distribution of irregular developing bands and radiating sectors of highly fluctuating widths. We also noticed high variability in the distribution of purple from one specimen to another. The endogenous pigmentation of oyster shells appeared to be under genetic control. Indeed, selective breeding strategies by inheritance lead to the production of oyster shells of the desired colours^38–42^. Recently, a locus devoted to shell purple pigmentation has been highlighted in *C. gigas*, which again attests to the endogenous origin of this pigmentation^43^. Biosynthesized according to specific metabolic pathways, the translation of the associated genes may subsequently be affected by exogenous factors such as salinity, temperature, sun or diet^44^.

In the present study, we have demonstrated the occurrence of a set of porphyrins in different parts of the edible oyster *C. gigas*. These data shed new light on the possible correlation between the purple pigmentation of the outer shell (common colour), the darkness of the adductor muscle scar (hidden colour) and the secretions of the mantle. The identification of uroporphyrin and derivatives was demonstrated in each case. Conversely, none of these porphyrins were detected in viscera, suggesting the MEE-specific accumulation or secretion of porphyrins related to purple and dark shell pigmentations. This shell pigmentation process fundamentally differs from what can be observed in other aquatic animals, such as *Artemia,* where its pigmentation derives from the ingestion of asthaxanthin-rich algae^45^.

The exclusive deposition of uroporphyrin and derivatives in shell purple patterns and dark AMS, accumulated in the MEE, raises the question of their biological origin, role and function for the living animal. Shell colour is a phenotypic trait in bivalves, which in many cases is evolutionarily designed according to inherited biosynthetic pathways, such as the highly conserved haem pathway in animals. As recently demonstrated in the case of marine snail, carboxylic acid porphyrins contribute to the pink-red and yellow–brown colours of shells^20^. These porphyrins are considered as side products of the haem biosynthetic pathway^3^. Haem is a well-known porphyrin complexed with Fe(II), able to transport oxygen indispensable for cellular aerobic respiration. This biosynthetic pathway consists of a succession of reactions enzymatically driven. The non-enzymatic side path leads to the production of uroporphyrin and derivatives by the oxidation of uroporphyrinogen I and III (Supplementary Fig. S10).

In the present case study, a similar process could occur, with oyster shell porphyrins may derive from the non-enzymatic oxidation of uroporphyrinogen I and III associated with the cellular respiration of the shell forming tissue, especially in periods of high loading. This assumption is strongly supported by the recent elucidation of the *C. gigas* genome where genes associated with enzymes of the haem pathway are expressed, such as ferrochelatase, protoporphyrinogen oxidase, or delta-aminolevulinic acid dehydratase^46^. If the synthetic pathways of these pigments begin to emerge, the mechanism of their deposition in the shell still remains an opened issue. The answer may lie in the mineralization front. From a material science point of view, it has been reported that carboxylate-containing molecules are possible structure directing agents that can preferentially adsorbed on calcium carbonate and orient the crystal growth^47^. Also, monolayers of an amphiphilic porphyrin bearing carboxylic acid groups are reported to nucleate the (001) plane of calcite, suggesting a possible epitaxial relation between the carboxylic acid groups and the (001) face of calcite related to the bidentate interaction with the carboxylic acid groups of porphyrins^48^. Therefore, a possible scenario may be the production of uroporphyrin and its derivatives resulting from the oxidation of uroporphyrinogen I and III, especially during periods of high activity of the respiratory cells from the contractile tissues of MEE and adductor muscle. These porphyrins being then integrated into the shell structure by the ionic binding between carboxylate groups and Ca^2+^. Uroporphyrin and derivatives are necessarily present in the shell mineralization matrix, possibly as a mineralization cofactor, in addition to achieving, supporting or catalysing one or more biological functions. Another option would simply be the result of an accidental deposition in the shell. Porphyrins would be evacuated during shell formation as metabolic wastes resulting from the respiration of cells constituting the mantle and adductor muscle.

Finally, we have noticed that among juvenile and adult edible oysters, purple is a common colour of the outer shell but not systematically found. During the early stages of mineralization when the shell is thin, shell colouration may provide protection from sunlight, especially at low tide. This raises the question of a more significant, or even singular, translation of genes involved in the production of carboxylic acid porphyrin in certain *C. gigas* compared to specimens with non-purple shells. While all have the same genome in common, we can hypothesis that some have pseudogenes or inactive genes that are non-functional and therefore unable to lead to the expression of a particular protein. Is this observation the result of a general evolution in edible oysters, of a primitive/ancestral trace or of an adaptation to a particular environment, or is it the result of a simple non-natural genetic selection carried out by breeders in search of oysters with purple shells for their consumers? The discovery of a rare and natural source of porphyrins offers here an unprecedented opportunity to give more added value to purple waste oyster shells. Although extraction yield and purification still need to be improved, the extraction of porphyrins may have significant potentials for biotechnology or photoactivation applications^49,50^.

## Materials and methods

### Sample collection

Samples of *C. gigas* were collected and supplied by TARBOURIECH-MEDITHAU (Marseillan, France, GPS coordinates: 43.382127, 3.555193, August 2017). Shell fragments were collected on living adult oysters (2 years old), it consists of pieces of few cm^2^ with a thickness ~ 1–2 mm, either white, partially or fully purple coloured. Shell fragments were sorted in two classes according to their colour, namely white and purple (WOS and POS respectively). Valves with dark adductor muscle scar (AMS) were collected from wasted adult oyster shells. Viscera and mantle edge epithelium tissues were obtained from ten 10 adult *C. gigas* oysters resulting from the daily determination of flesh content, performed by TARBOURIECH-MEDITHAU, in accordance with the inter-branch agreements on oyster packaging. Tissues were placed in glass bottles and kept in the dark at − 18 °C and transported to the laboratory. Samples were carefully rinsed with deionized water, freeze-dried and stored in the dark at − 21 °C before investigation.

### Sample decontamination

POS, WOS and valves with dark AMS were extensively rinsed with tap water followed by immersion in successive baths of NaOCl_1%_ and deionized water, assisted by sonification (1:10 wt./V, 120 min). Further rinses were performed with deionized water. Samples were finally dried in oven (overnight, 40 °C) and stored in the dark at 25 °C before use.

### Structural characterization by scanning electron microscopy

Dark AMS from decontaminated valves were prepared with cutting pliers and the outer shell layers were removed using a DREMEL 3,000 polisher. Decontaminated POS, WOS and dark AMS were fixed on supports and metalized with Pt by Ar plasma-enhanced chemical vapour deposition (QUORUM TECHNOLOGIES), or analysed as drawn. Images were recorded with a field emission scanning electron microscope HITACHI S4800 equipped with a secondary and a backscattered electrons detector (accelerating voltage from 0.1 to 15 kV, resolution of 1 nm at 15 kV, maximum magnification 800,000×). The insoluble residue was fixed on a support and metallized with Pt for surface elemental composition achieved with a field emission gun detector (OXFORD INSTRUMENTS X-MAX SDD) coupled with a scanning electron microscope ZEISS EVO HD15 equipped with a secondary and backscattered electrons detector (resolution of 1.9 nm at 30 kV under ultra-high vacuum).

### Solid state fluorescence

POS, WOS, dark AMS and *Pinctada radiata* shells (author’s personal collection) were exposed, in the dark, to a UV LED monochromatic light (395–400 nm, 10 W, model CHX-FL-A-10 W).

### Identification of shell tetrapyrroles by photoluminescent emission and fluorescence spectroscopy

Decontaminated POS (30 g), WOS (30 g) and powdered dark AMS (97 g) were dissolved in 1 M aqueous HCl under magnetic stirring (1:20 wt./V, 30 min, 700 RPM). The acidic extracts, namely ASM^POS^, ASM^WOS^ and ASM^AMS^, were obtained after filtration on a glass sintered filter (POR 4) filled with Fontainebleau sand. Photoluminescence was investigated by exposition, in the dark, of ASM^POS^, ASM^WOS^ and ASM^AMS^ to the UV LED monochromatic light. Excitation and emission spectra of ASM^POS^ and ASM^WOS^ (0.5 mg/mL) were recorded on a fluorescence spectrometer (FS920, EDINBURGH INSTRUMENTS) equipped with a 450 W continuous xenon arc lamp as the excitation source for steady-state photoluminescence measurements using quartz cells with 10 mm excitation path length. Excitation spectra were recorded at an emission wavelength of 656 nm (from 348 to 626 nm with a step of 0.1 nm, 26.7 °C). Emission spectra were recorded at an excitation wavelength of 405 nm (from 425 to 790 nm with a step of 0.1 nm, 26.8 °C).

### UV–vis absorption spectroscopy

Absorption spectra of ASM^POS^ (5 mg/L), ASM^WOS^ and ASM^AMS^ (50 mg/L) were recorded from 200 to 800 nm using UV-1800 SHIMADZU spectrophotometer (10 mm optical path length). Appropriate auto zero on solvent blank (1 M aqueous HCl) was performed before each measurement.

### Identification of shell uroporphyrin and derivatives by RPLC-HRMS

Identifications were performed using a RPLC-HRMS system (WATERS ALLIANCES UPLC SYNAPT G2-S) in positive electrospray ionization with a *m/z* range of 50 to 3,000. High resolution mass spectra were recorded with a ZQ spectrometer fitted with Micromass Q-Tof spectrometer operating at capillary voltage of 3 kV and cone voltage of 30 V, using phosphoric acid as an internal standard. MASSLYNX software (version V4.1) was used for instrument control and data processing. Separations were carried out using a 150 × 2.1 mm KINETEX 2.6 μm EVO C18 100 Å reverse stationary phase, operating at 30 °C with a constant flow rate of 0.5 mL/min. Compounds were separated using linear gradient and isocratic systems of ultrapure water (0.055 μS/cm) and acetonitrile HPLC grade containing 0.1% formic acid (0 to 20% acetonitrile in 3 min followed by an isocratic elution with 20% acetonitrile for 17 min, followed by 20 to 50% acetonitrile in 12 min and 50 to 100% acetonitrile in 0.1 min). Analysed samples consist of ASM^POS^, ASM^WOS^ (50 mg/L each) and a chemical standard of uroporphyrin I dihydrochloride (SANTA CRUZ BIOTECHNOLOGY, batch H1219), dissolved in 1 M aqueous HCl. A blank of 10 μL of 1 M aqueous HCl was injected before each sample (10 μL injected).

### Identification of uroporphyrin in mantle edge epithelium by RPLC-HRMS

Freeze-dried tissues (MEE and viscera, 1 g each) were crushed in a mortar and homogenized in 1 M aqueous HCl under magnetic stirring (1:20 wt./V, 60 min, 700 RPM). The acidic extracts, namely ASM^MEE^ and ASM^VOT^, were obtained after filtration on a glass sintered filter (POR 4) filled with Fontainebleau sand. The identification of uroporphyrin and chlorophyll catabolites were achieved using RPLC-HRMS previously described. A blank of 10 μL of 1 M aqueous HCl was injected before each sample analysis in order to avoid-cross-contamination (10 μL injected).

### Concentration of shell porphyrins by precipitation with hydrofluoric acid (HF)

Concentration of ASM^POS^ was conducted by first reducing the volume to approximately 125 mL by evaporation (16 mbar, 35 °C) followed by the addition of 27.6 M aqueous HF in excess. After 30 min under gentle stirring, the sample was centrifuged (20 min, 4,400 RPM). The supernatant was then concentrated to dryness (atmospheric pressure for 7 days at 25 °C in the dark followed by vacuum evaporation at 35 °C). The solid residue was suspended in ultrapure water (40 mL, 0.055 μS/cm), stirred with a vortex stirrer and centrifuged (20 min, 4,400 RPM). The solid material resulting from centrifugation was washed a second time with ultrapure water (15 mL, 0.055 μS/cm) and freeze-dried (extraction yield of 0.1%). The entire process was also applied to ASM^AMS^ (extraction yield of 0.004%). The extracts were stored in the dark at 4 °C until RPLC-HRMS analysis previously described and tandem mass fragmentation with 10 μL samples (10 mg/mL in 1 M aqueous HCl).

### Chemical confirmation of uroporphyrin I by NMR spectroscopy

Purification of major porphyrins found in concentrated ASM^POS^ was carried out on a semi-preparative UHPLC-DAD system in the UV–vis range of 200–800 nm (THERMO SCIENTIFIC DIONEX ULTIMATE 3,000). Separation was performed using a 250 × 10 mm KINETEX 5 μm C18 100 Å reverse stationary phase, operating at 25 °C with a constant flow rate of 2.0 mL/min. Isolation was performed using four gradients, followed by an isocratic elution and a final gradient system of ultrapure water (0.055 μS/cm) and acetonitrile HPLC grade containing 0.1% formic acid (0 to 27% acetonitrile in 3.5 min followed by 27 to 35% acetonitrile in 14.5 min, followed by 35 to 50% acetonitrile in 1 min, followed by 50 to 80% acetonitrile in 2 min; isocratic elution was conducted with 80% acetonitrile in 1 min and the final gradient system with 80 to 100% acetonitrile in 5 min). Sufficient amount of uroporphyrin was collected and freeze-dried. The isolated uroporphyrin and the chemical standard of uroporphyrin I (1 mg) were dissolved in 600 μL of DMSO-d6 (SIGMA-ALDRICH). ^1^H spectra were recorded from -5 to + 13 ppm (64 scans, BRUKER AVANCE III HD 400 MHz).

## Supplementary information

Supplementary information

## Data Availability

The data that support the findings of this study are available from the corresponding authors on reasonable request.
