# Supplementary material for: Chemical evidence of rare porphyrins in purple shells of Crassostrea gigas oyster
Source: Sci Rep. 2020 Jul 22;10:12150. doi: 10.1038/s41598-020-69133-5 (PMC7376061; doi:10.1038/s41598-020-69133-5)
Supplement: Supplementary file 1 — Supplementary information [file 41598_2020_69133_MOESM1_ESM.pdf]

Chemical evidence of rare porphyrins in purple shells of *Crassostrea gigas* oyster

Michel Bonnard[a,b], Sonia Cantel[a], Bruno Boury[c,*], Isabelle Parrot[a,*]

[a] IBMM, CNRS, ENSCM, Univ Montpellier, Montpellier, France

[b] Tarbouriech-Médithau, Marseillan, France

[c] Institut Charles Gerhardt, CNRS, ENSCM, Univ Montpellier, Montpellier, France

Supplementary information

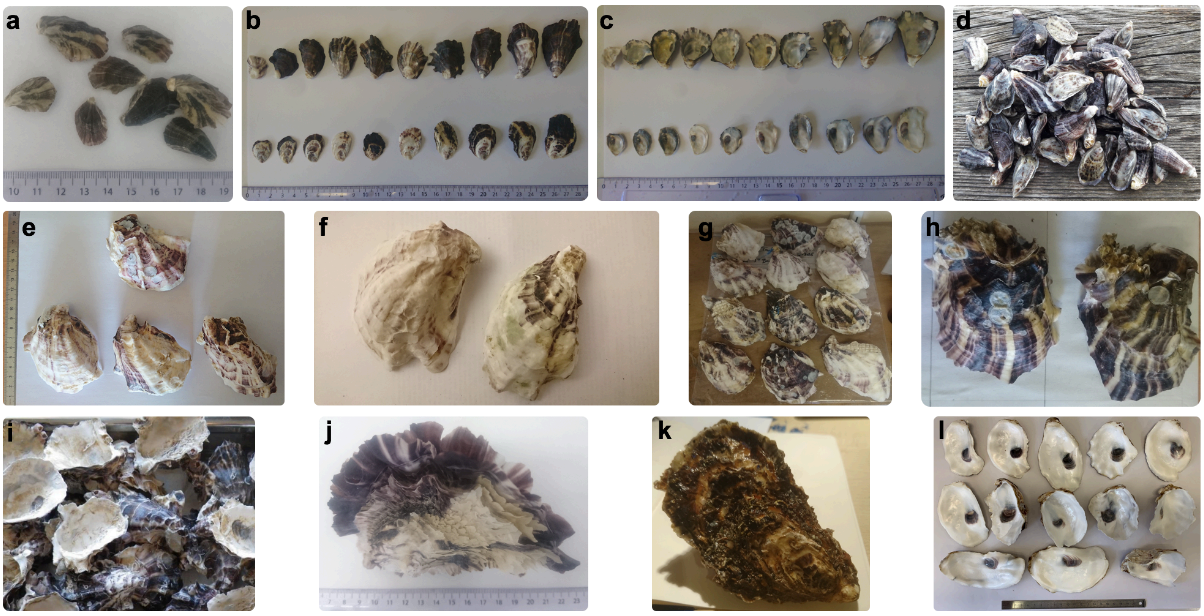

Supplementary Fig. S1 Photographs of purple patterns distribution in juvenile and adult *C. gigas* shells collected from France and Italy. a Decontaminated shell of juvenile oysters bred in Bouin, France (supplied in August 2017). **b-c** Decontaminated shells of juvenile oysters farmed in Scardovari, Italy (supplied in August 2017). **d** Juvenile oysters farmed in Marseillan, France (December 2018). **e-f** Decontaminated shells of adult oysters collected in Ile d'Oleron, France (collected in December 2018). **g-j** Decontaminated shells of adult oysters collected in Marseillan, France (collected in January 2017). **k** Adult oyster farmed in Bouzigues, France (collected in November 2019). **l** Decontaminated valves with dark AMS farmed in Marseillan, France (collected in August 2017).

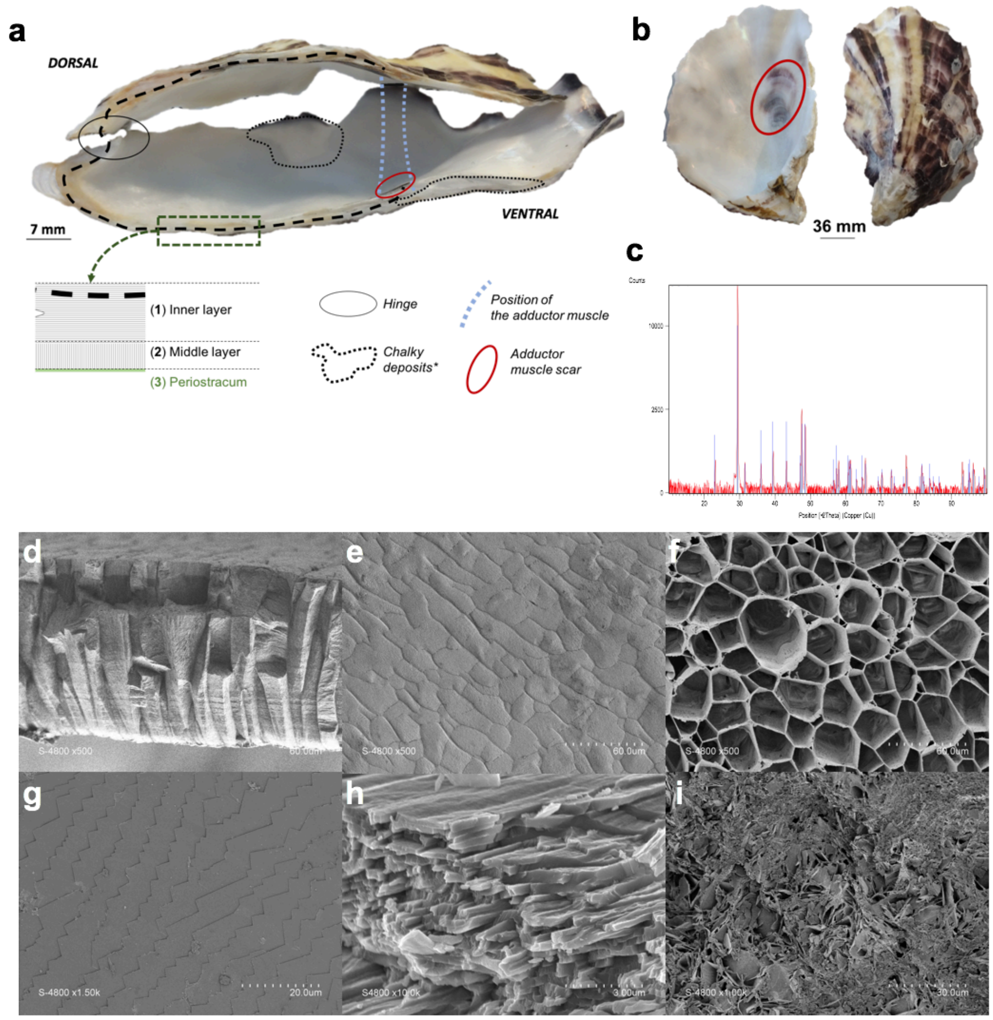

Supplementary Fig. S2 Schematic of the structure of *C. gigas* shell. a Cross-section with a scheme of the shell layout. **b** Dark AMS of a ventral vale. **c** XRD powder diffractogram of decontaminated and powdered *C. gigas* shell (red) superimposed with a reference diffractogram of calcite. **d-e** Characteristic SEM images of decontaminated outer prismatic layer, generally observed on only one of the valves (cross-section and top view, respectively). **f** Characteristic SEM image of the interprismatic organic matrix obtained after dissolution of a purple prismatic fragment in 1M aqueous HCl. **g-h** Characteristic SEM images of decontaminated inner foliated layer (cross-section and top view, respectively). **i** Characteristic SEM image of decontaminated chalky structure interrupting the foliated inner layer.

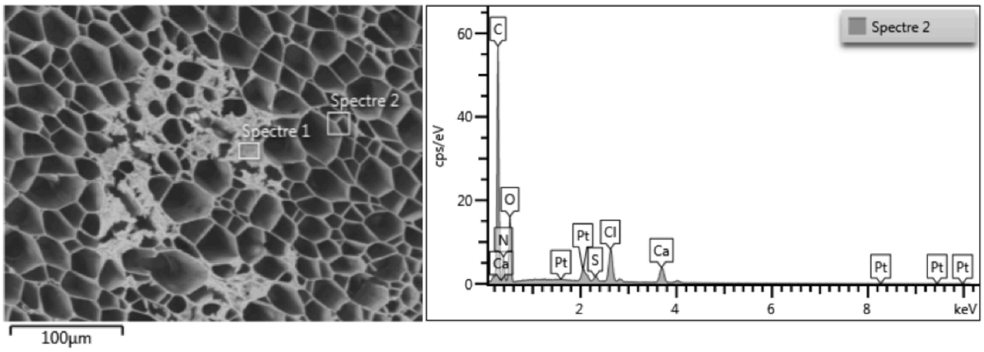

Element	Atomic %
C	61.6
N	17.2
O	16.1
S	0.3
Cl	2.6
Ca	2.2

Supplementary Fig. S3 Elemental analysis of the interprismatic organic matrix obtained after dissolution of a purple prismatic fragment in 1M $HCl_{(aq)}$. Residual undissolved calcium carbonate contributes to the Ca and C content. HCl contributes to the Cl content. Pt observed from spectrum 2 is due to the Pt-metallization of the sample, it has been removed from the calculation of the atomic percentage.

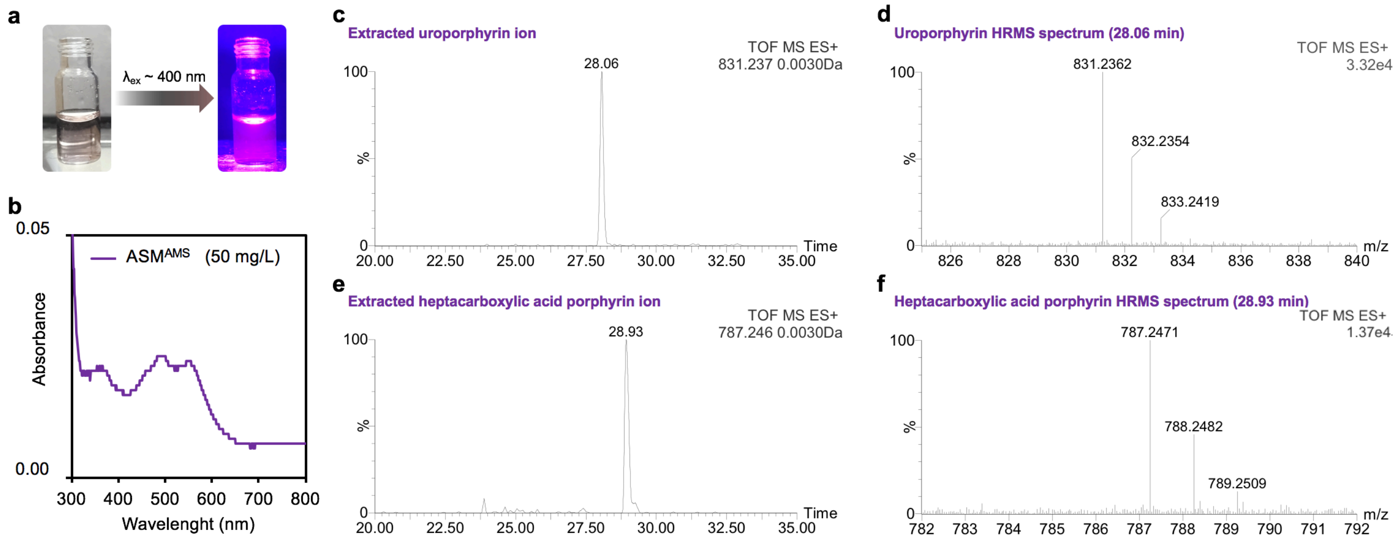

Supplementary Fig. S4 Photophysical properties of ASM^{AMS} and chemical investigation by RPLC-HRMS. a Photoluminescence of ASM^{AMS}. **b** UV-vis absorption spectrum of ASM^{AMS}. **c-d** Chromatogram of extracted uroporphyrin I or III ion detected in concentrated ASM^{AMS} with the corresponding high resolution mass spectrum. **e-f** Chromatogram of extracted heptacarboxylic acid porphyrin I or III ion detected in concentrated ASM^{AMS} with the corresponding high resolution mass spectrum.

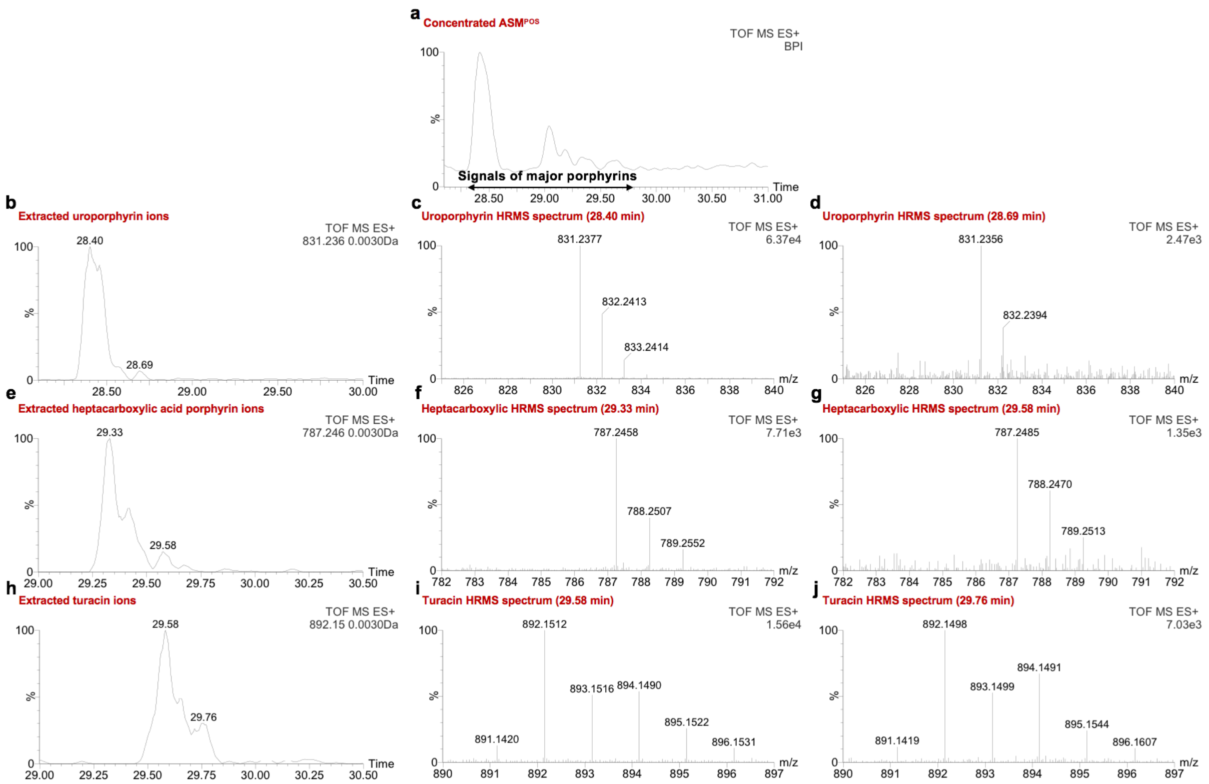

Supplementary Fig. S5 Identification of major porphyrins by RPLC-HRMS in concentrated ASM^{POS} in electrospray positive ionization mode. a Elution profile of major porphyrins found in concentrated ASM^{POS}. **b-d** Chromatogram of extracted uroporphyrin ions with corresponding high resolution mass spectra. **e-g** Chromatogram of extracted heptacarboxylic acid porphyrin ions with corresponding high resolution mass spectra. **h-j** Chromatogram of extracted turacin ions with corresponding high resolution mass spectra.

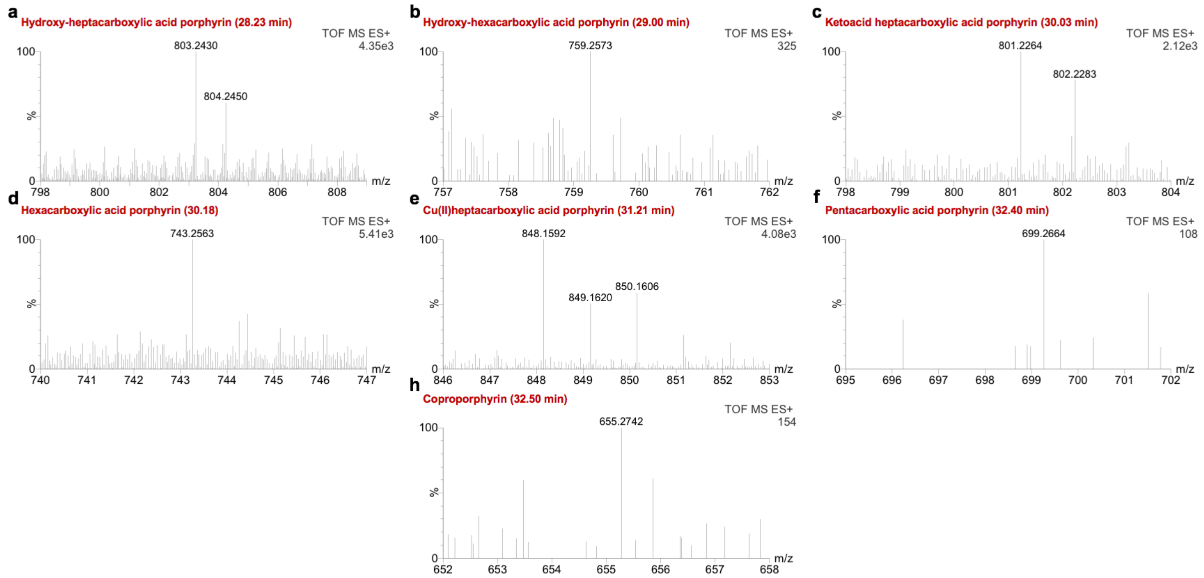

Supplementary Fig. S6 High resolution mass spectra of minor porphyrins identified in concentrated ASM^{POS}. a-h High resolution mass spectra of hydroxy-heptacarboxylic, hydroxy-hexacarboxylic, ketoacid heptacarboxylic, hexacarboxylic, Cu(II)heptacarboxylic, pentacarboxylic acid porphyrins and coproporphyrin, respectively.

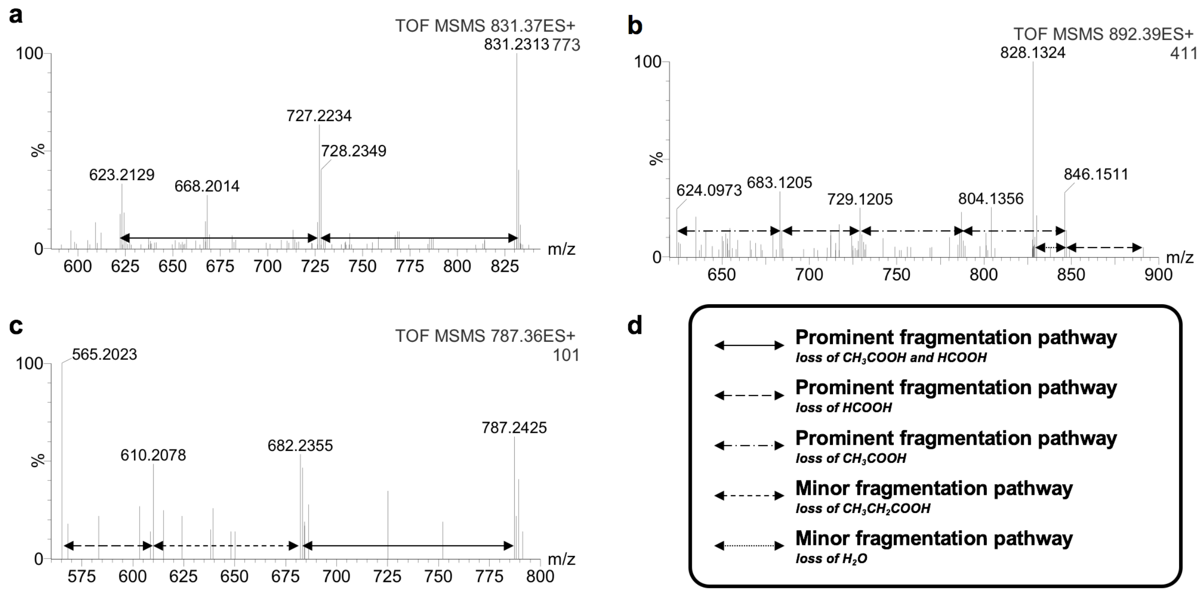

Supplementary Fig. S7 Chemical confirmation of major porphyrins found in concentrated ASM^{POS} by tandem mass fragmentation spectroscopy. a-c Tandem mass fragmentation spectra of uroporphyrin, turacin and heptacarboxylic acid porphyrin I or III ions, respectively. **d** Fragmentation pathways of carboxylic acid porphyrins.

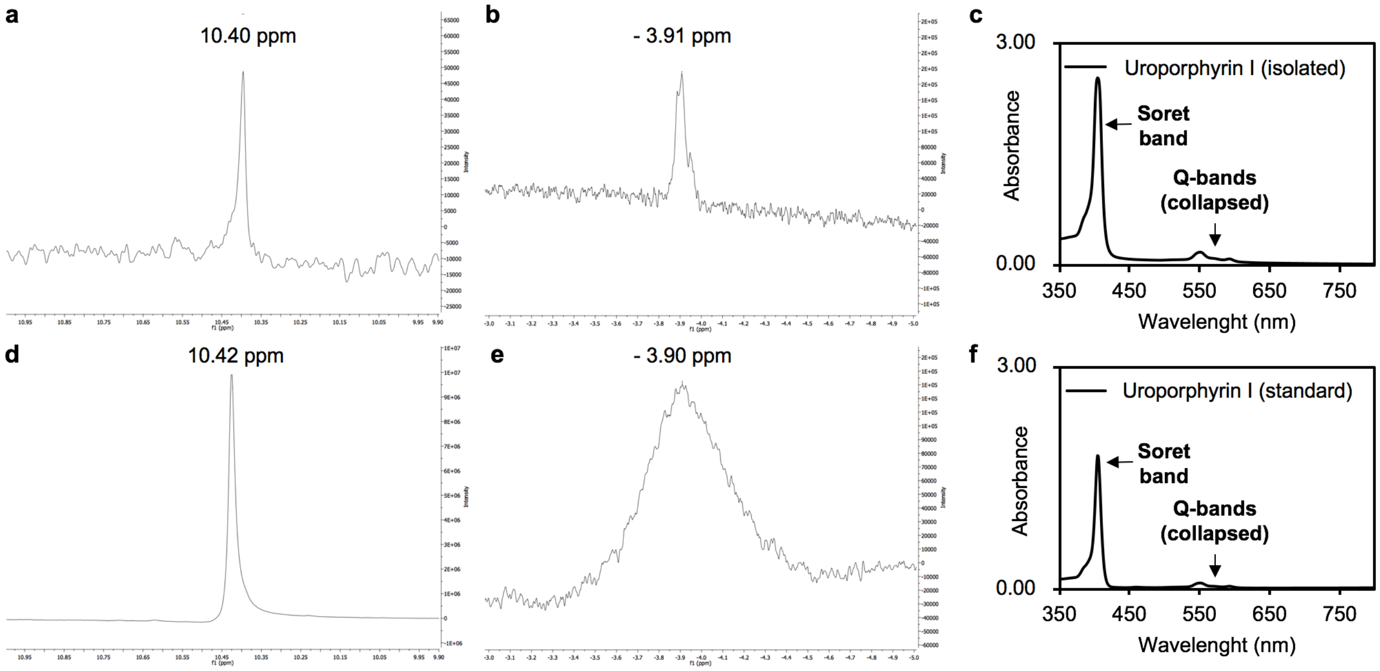

Supplementary Fig. S8 Characteristic ^{1}HMR chemical shifts and UV-vis spectra of uroporphyrin I isolated from the concentrated ASM^{POS} in comparison with the chemical standard of uroporphyrin I. **a-b** Proton chemical shifts of COOH and NH of uroporphyrin I isolated from concentrated ASM^{POS}. **c** UV-vis absorption spectrum of uroporphyrin I isolated from concentrated ASM^{POS}. **d-e** Proton chemical shifts of COOH and NH of uroporphyrin I chemical standard. **f** UV-vis absorption spectrum of uroporphyrin I chemical standard.

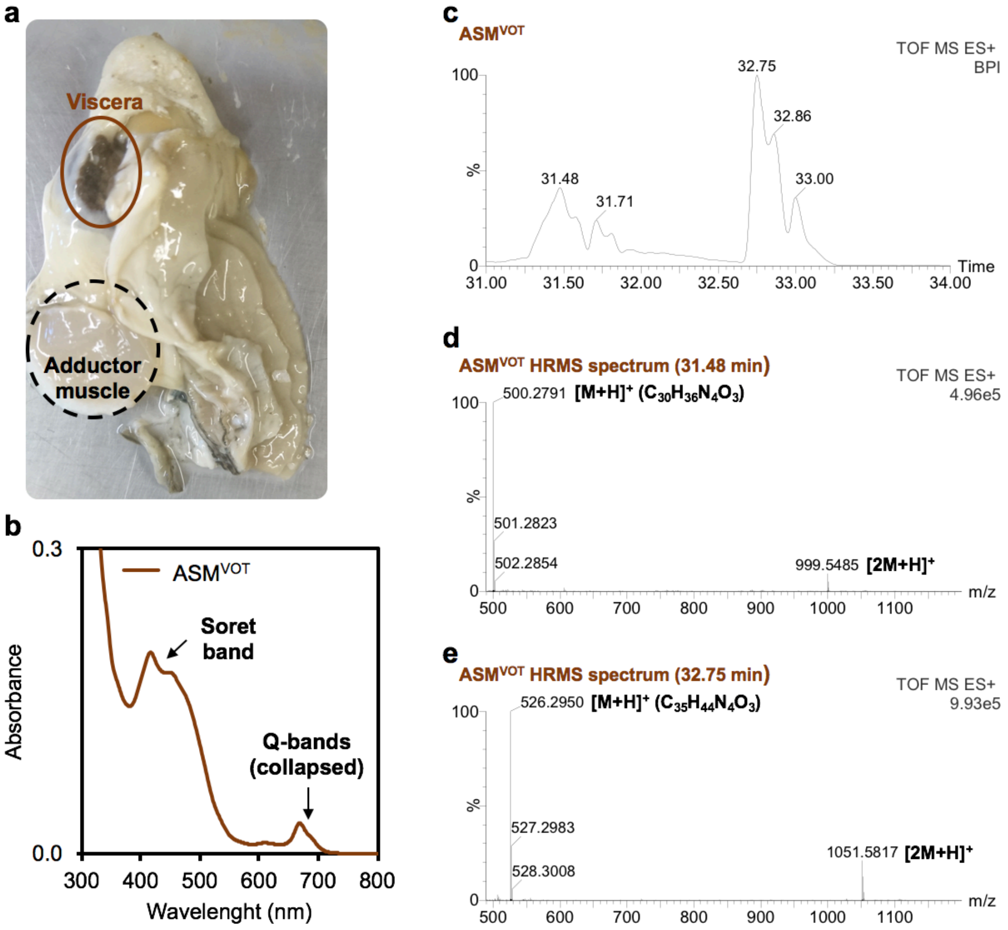

Supplementary Fig. S9 Identification of chlorophyll catabolites in viscera of *C. gigas*. a Schematic figure of the brown viscera after collection of the mantle edge epithelium (MEE). **b** UV-vis absorption spectrum of ASM^{VOT}. **c-e** Chromatogram of ASM^{VOT} showing signals of chlorophyll catabolites with corresponding high resolution mass spectra, respectively.

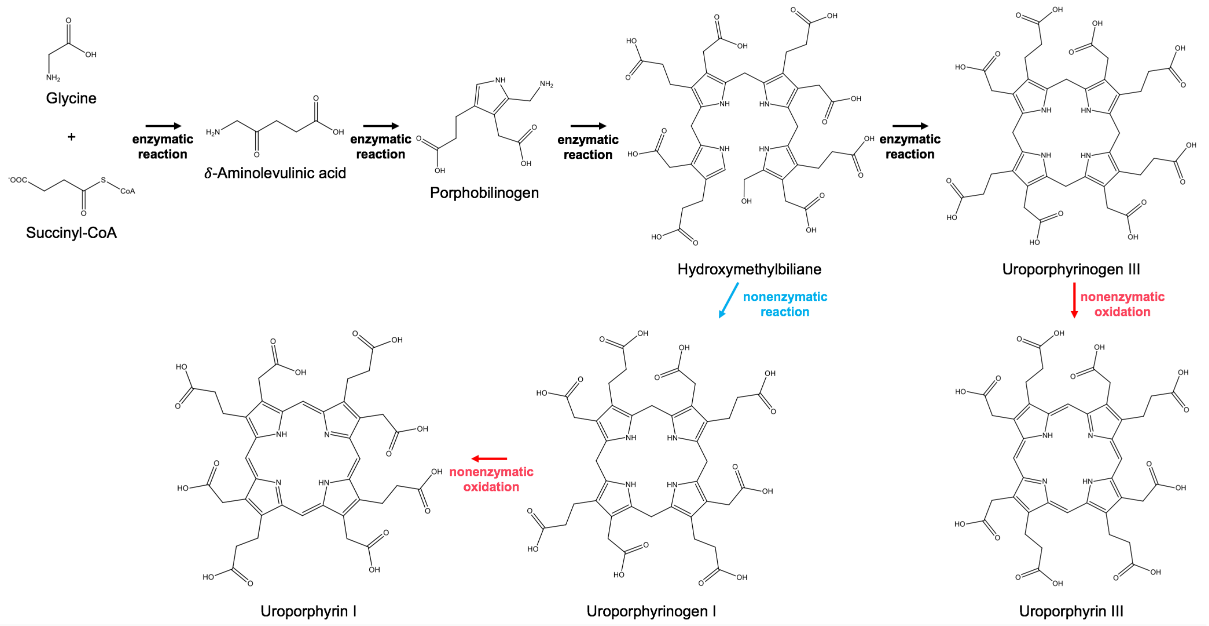

Supplementary Fig. S10 Schematic representation of the fourth first enzymatic-catalysed reactions of the haem biosynthetic pathway including the non-enzymatic side path.
